# Anti-quorum sensing evaluation of methyleugenol, the principal bioactive component, from the *Melaleuca bracteata* leaf oil

**DOI:** 10.3389/fmicb.2022.970520

**Published:** 2022-08-22

**Authors:** Wenting Wang, Xiaojie Lin, Huixiang Yang, Xiaoqin Huang, Lei Pan, Shaohua Wu, Chao Yang, Liaoyuan Zhang, Yongyu Li

**Affiliations:** ^1^College of Horticulture, Fujian Agriculture and Forestry University, Fuzhou, China; ^2^Institute of Natural Products of Horticultural Plants, Fujian Agriculture and Forestry University, Fuzhou, China; ^3^College of Life Sciences, Fujian Agriculture and Forestry University, Fuzhou, China

**Keywords:** quorum sensing inhibitor (QSI), methyleugenol, *Melaleuca bracteata* EO, *Chomobacterium violaceum* ATCC31532, virulence factors, signal molecule (C6-HSL)

## Abstract

Quorum sensing (QS) is a cell-to-cell communication in bacteria that couples gene expression through the accumulation of signaling molecules, which finally induce the production of several virulence factors and modulate bacterial behaviors. Plants have evolved an array of quorum sensing inhibitors (QSIs) to inhibit the pathogens, of which aromatic compounds are widely recognized. The essential oil of *Melaleuca bracteata* was found to exhibit anti-quorum sensing activity, and its principal bioactive component, methyleugenol (ME), had been isolated in our previous study. Here, ME interfered effectively with the QS-regulated processes of toxin secretion in *Chomobacterium violaceum* ATCC31532, resulting in strong inhibition of QS genes, *cviR*, *cviI*, *vioA-E*, *hmsHNR*, *lasA-B*, *pilE1-3*, and *hcnABC*, leading to impaired virulence, including violacein production, biofilm biomass, and swarming motility. The accumulation of the signal molecule (N-hexanoyl-DL-homoserine lactone, C6-HSL) in *C. violaceum* declined upon treatment with ME, suggesting an inhibition effect on the C6-HSL production, and the ME was also capable of degrading the C6-HSL *in vitro* assay. Molecular docking technique and the consumption change of exogenous C6-HSL in *C. violaceum* CV026 revealed the anti-QS mechanism of ME consisted of inhibition of C6-HSL production, potentially *via* interaction with CviR and/or CviI protein. Collectively, the isolated ME, the principal active components of *M. bracteata* EO, exhibited a wide range of inhibition processes targeting *C. violaceum* QS system, which supports the potential anti-pathogenic use of *M. bracteata* EO and ME for treatment of pathogen contamination caused by bacterial pathogens.

## Introduction

With the increasing number of drug-resistant pathogens worldwide, the novel strategies to fight the pathogens are needed. The introduction of “anti-virulence” therapeutics has been expected to be an effective strategy to prevent or at least minimize the selective pressure toward resistance development (Wu et al., 2014; Joshi et al., 2016a). A promising approach has been developed to interfere with the bacterial pathogenicity, commonly known as quorum sensing (QS) quenching. The QS system is a cell-to-cell communication mechanism that usually involves the small, diffusible signal molecules termed autoinducers (AIs) (Chong et al., 2011), which can be adjusted by the bacterium population density. A threshold of population density results in forming a complex (AI binds to its cognate receptor), which will in turn regulate the multitudinous gene expression, particularly those responsible for virulence (Morohoshi et al., 2008; Packiavathy et al., 2014). Interruption of QS quenching (QSQ) is focused on blocking bacterial pathogenesis without killing bacteria or inhibiting their growth.

*Chromobacterium violaceum* is an gram-negative pathogenic bacteria found in soil and water in tropical and subtropical areas (Yang and Li, 2011). It is occasionally pathogenic in immunocompromised individuals or children where it may cause diarrhea; though not involved in human infections, this bacterium is being viewed as an emerging pathogen (Duran et al., 2010). The whole genome sequence of *C. violaceum* ATCC12472 was revealed in 2003 and revealed the presence of QS-relative genes (de Almeida et al., 2003). *C. violaceum* has become a model organism for studying the bacterial communication in gram-negative bacteria and harbors sole QS mechanisms that depend on the type of the released signaling molecule (Kothari et al., 2017). In the QS system of *C. violaceum*, N-Hexanoyl-DL-homoserine lactone (C6-HSL), an acyl homoserine lactones (AHLs), is produced and detected by the CviI/CviR system. CviR genes encode CviR regulator proteins, while cviI genes encode the CviI synthase necessary for the synthesis of C6-HSL. Altogether, a complex of violacein, biofilm formation, exopolysaccharides (EPS), motility movement, and other virulence factors is activated when C6-HSL binds to the CviR protein (Duran et al., 2016).

Many naturally derived compounds have been reported to exhibit the inhibitory property on bacterial virulence expression. The first quorum sensing inhibitor (QSI), efficient in reducing bacterial virulence determinants, is a halogenated furanone isolated from the red marine alga *Delisea pulchra* (Chong et al., 2011). Subsequently, more QSIs have been found and isolated in higher plants (Kerekes et al., 2013). Malabaricone C from *Myristica cinnamomea* exhibits anti-QS activity against *C. violaceum* CV026 and *Pseudomonas aeruginosa* PAO1 (Chong et al., 2011). Plant phenolic acids affect the pathogenicity of soft rot enterobacteria (Joshi et al., 2015, 2016b). The components of the flavonoid family, such as rutin, myricetin, 3-O-rutinoside, and kaempferol-3-O-rutinoside from *Pistacia atlantica*, have high anti-QS activities against *P. aeruginosa* PAO1 (Kordbacheh et al., 2017). Eugenol and linalool could affect the synthesis of QS proteins like LasA and LasB as well as virulence factors such as pyocyanin and rhamnolipids, which seriously hamper the formation of biofilm (Lahiri et al., 2021). *Mentha piperita* essential oil at sub-MICs strongly interfered with AHL regulated virulence factors and biofilm formation in *P. aeruginosa* and *Aeromonas hydrophila* (Husain et al., 2020). More studies were elucidated on the inhibitory mechanism of QSIs on the pathogens based on the detection of the major virulence determinate and the molecular docking of the specific ligand-protein interaction. Therefore, more experimental studies need to be provided.

Our group embarked on a study to discover the components of anti-QS activity from *Melaleuca bracteata*, which is a tree with yellow leaves in the Myrtle family Myrtaceae. Originated from New Zealand, mainly in Australia, it was introduced and cultivated in China in 1999. *M. bracteata* is a precious aromatic plant with highly ornamental value and economic benefits. The obvious antibacterial activity and oxidation resistance of *M. bracteata* EO have been reported by many researchers (Li et al., 2018; Siddique et al., 2020). Its prominent physiological and biochemical activities were due to the production of an abundance of secondary metabolites such as phenolics, terpernoids, and polyacetylenes. Previous studies in our group found that *M. bracteata* leaf EO had an intensively inhibitory effects on *C. violaceum* QS (Wang et al., 2019a). However, it is still unclear which active components in *M. bracteata* leaf EO play an important role as QSI, which is not conducive in clarifying the inhibitory mechanism of *M. bracteata* leaf EO against the bacterial QS. This study was extended further to identify the effect of the principal active component, methyleugenol (ME), isolated from *M. bracteata* leaf EO (Not published), on QS dependent virulence in *C. violaceum* ATCC31532.

## Materials and methods

### Materials, medium, and growth conditions

*Chomobacterium violaceum* strains ATCC31532 and CV026, stored in the College of Horticulture at Fujian Agriculture and Forestry University (Fujian, China), were inoculated in LB broth and grown under conditions of 30°C, 150 rpm. CV026 is a mutant of the *C. violaceum* strain ATCC31532, lacking the autoinducer synthase cviI and requiring the exogenous C6-HSL to induce the violacein production. N-Hexanoyl-DL-homoserine lactone (C6-HSL) standard solution was purchased from Sigma-Aldrich (Shanghai, China); acridine orange and crystal violet were purchased from Solarbio (Beijing, China).

#### Plant material

*Melaleuca bracteata* leaves were harvested annually at Fujian Agriculture and Forestry University (Fujian, China) and identified by Professor Fangying Li in the College of Art and Landscape Architecture of Fujian Agriculture and Forestry University (Fujian, China).

### Determination of minimum inhibitory concentrations and growth curve

Doubling dilution method was applicable to test the minimum inhibitory concentrations (MIC) of ME (Zins et al., 2001). Briefly, 1% of *C. violaceum* (0.9 OD at 600 nm) was added to appropriate LB medium (100 μl) supplemented with 2-fold serially diluted ME, whose concentration ranged from 0.625 to 80‰ of equal final volume (100 μl) in 1.5 ml microcentrifuge and cultured for 24 h at 30°C. The OD600 was measured, and the MIC was viewed as the lowest concentration that inhibited completely the visible purple pigment production and did not affect the bacterial growth. All further experiments in this study were performed at sub-MIC concentrations of ME.

The 1% of *C. violaceum* (0.9 OD at 600 nm) was incubated in a 250-ml Erlenmeyer flask containing 20 ml of LB broth supplemented with ME (sub-MIC, 6% v/v), the mixing was cultured at 30°C under 150 rpm in a rotatory shaker. The growth of *C. violaceum* was monitored using OD600 over a period of 72 h, and it was further quantified by plating the cultures and counting the colony-forming units (CFU) at 12 and 24 h.

### Detection of violacein production

Overnight cultured 1% *C. violaceum* (0.9 OD at 600 nm) were added into glass tube containing 5 ml LB broth supplemented with various concentrations (sub-MIC, 6% v/v) of ME. The mixing liquid was incubated at 30°C for 12 h, and the violacein inhibition was measured using UV-visible spectrophotometry at OD595. The specific methods were performed following the method described (Khan et al., 2009). Each assay was performed in triplicate.

### Biofilm inhibition assay

The effect of ME on biofilm was performed by quantifying the biofilm biomass, using a 96-well microtiter dish with crystal violet (CV) for biofilm staining as described by the Microtiter Dish assay (O’Toole, 2011; Wang et al., 2019b). It was quantified by measuring absorbance at 550 nm in microplate reader and recorded as the absorbance of CV dye bound to the bacterial biofilm. Each assay was performed in eight replicates. To identify the ability of ME in disrupting biofilm, the assay was performed following the revised method described (Packiavathy et al., 2012). Briefly, the *C. violaceum* treated with different concentrations of ME (sub-MIC, 6% v/v) was developed in a 6-well plate with cover glasses 1 cm × 1 cm for 12 h, and the biofilm was stained with CV and acridine orange (Becker et al., 2009), then observed under the light microscope and the confocal laser scanning microscope (CLSM).

### Inhibition of performed biofilm

*Chomobacterium violaceum* biofilm incubated in a 96-well plate. Once biofilm formed, the suspension cultures were removed and the wells were washed with sterile water three times. A quantity of 200 μl of fresh LB medium supplemented with ME (sub-MIC, 6% v/v) was added to the wells (5% tween 80 serving as the control). The culture was incubated at 30°C for 24 h, then the biofilm was stained with CV and quantitated after solubilization of the dye with 30% acetic acid by reading the microplates at 550 nm.

### Swarming motility

An effort was determined to examine ME against the swarming and swimming motility of *C. violaceum*. The specific method was performed following the method described (Packiavathy et al., 2014). In swarming assay, 5 μl overnight cultured bacterium (0.9 OD at 600 nm) were point incubated in 6-mm filter paper at the center of the swarming motility medium consisting of 1% tryptone, 0.5% NaCl, 0.5% agar, and 0.5% D-glucose with various concentrations of ME (sub-MIC, 6% v/v). For swimming assay, 5 μl overnight cultured bacterium (0.9 OD at 600 nm) were point incubated in 6 mm-filter paper at the center of the swarming motility medium consisting of 1% tryptone, 0.5% NaCl, and 0.3% agar with various concentrations of ME (sub-MIC, 6% v/v). Then, the plate was incubated at 30°C in an upright position for 16 h.

### The effect of methyleugenol on C6-HSL in *Chomobacterium violaceum*

The detection of C6-HSL was qualitatively and quantitatively determined by the CV026 biosensor and gas chromatograohy (GC), respectively. The procedures for extraction and detection were as described in the previous study (Wang et al., 2019a). The *C. violaceum* was grown in 20 ml LB broth for 12 h with or without ME (sub-MIC, 6% v/v). Then, C6-HSL extracts were prepared for further assay by GC detector and biosensor CV026. To study the potential degradation of C6-HSL by ME, adding a known synthetic standard (C6-HSL) to 20 ml LB broth treated with or without ME (sub-MIC, 6% v/v) was incubated for 0, 6, 12, and 24 h. The C6-HSL concentration was detected by GC detector.

Furthermore, the CV026 was grown in 20 ml LB broth for 12 h with or without ME (sub-MIC, 6% v/v) supplemented with the exogenous C6-HSL. The C6-HSL concentration was detected by GC. The consumption rate (%) was calculated, and the formula was as follows:


(1) Consumption (%)=(CI-CR)/CT× 100%

where CI is the initial concentration of exogenous C6-HSL, and CR is the concentration of control and treatment groups (incubated for 12 h).

### Gene expression analysis

Notably, 1% *C. violaceum* (OD600, 0.9) were incubated in 20 ml LB containing or not containing a range of ME concentration (sub-MIC, 6% v/v). Cultures were grown for 12 h, the cells were harvested by centrifugation (12,000 × *g*, 2 min), and the supernatant was discarded. Total RNA was extracted using an RNAprep Pure Cell/Bacteria Kit (Code No. DP430, TIANGEN, China), according to the manufacturer’s guidelines. The RNA was used for reverse-transcription, using Transcript One-step gDNA Removal and cDNA Synthesis SuperMix (Transgen, China). The oligonucleotide primers already used in the previous study were also used here to evaluate the level of transcript for the selected genes (Wang et al., 2019a). RT-qPCR was performed using Realtime PCR Master Mix (SYBR Green, Transgen, China) and the procedure was as follows: two steps at 94°C for 30 s and 40 cycles at 94°C for 5 s, and 60°C for 30 s. The calculated cycle threshold (CT) of each gene was normalized to the CT for rpoD amplified from the corresponding sample. The RT-qPCR was performed in Light cycler 96 (LightCycler^®^ 96, Roche Company, Switzerland). Fold changes in gene expression were calculated according to the 2-ΔΔCT method.

### Molecular docking

The molecular docking was used to explore the binding affinity of the active compound to the transcription factor CviR. The 3D structure of the ME molecule was downloaded from the ZINC database (the ZINC ID of the ligand ME was ZINC000000388674), and the crystal structure of CviR containing C6-HSL molecule was obtained from the website of RCSB PDB (PDB ID: 3QP1). Docking was then performed using Molsoft ICM with default parameters according to its protocol (Ismail et al., 2010), and the crystal structure of CviR ligand-binding domain bound to the native ligand C6-HSL was used as a positive control.

### Statistical analysis

All experiments were performed at least in triplicates, and all data were analyzed using the SPSS 19.0 software and presented as mean values and standard deviation. Differences with *P* < 0.05 were considered statistically significant.

## Results

### Minimum inhibitory concentrations detection of methyleugenol and growth curve analysis

**FIGURE 1:**
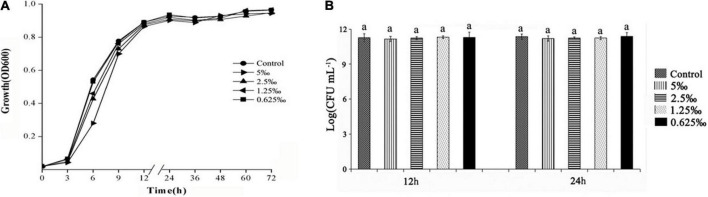
Effect of methyleugenol (ME) on growth of *Chomobacterium violaceum*. Growth curves of *C. violaceum* treated with ME at the varying concentrations of sub-MIC (5, 2.5, 1.25, and 0.625‰). **(A)** The control had no ME, **(B)** CFU of *C. violaceum* treated with ME for 12 and 24 h.

The high MIC would restrict the infection ability of bacteria. The MIC of ME was determined using the doubling dilution method with the concentration varying from 80 to 0.625‰. The MIC of ME was 10‰ for *C. violaceum*. Furthermore, the growth curve was evaluated at sub-MIC concentrations (5, 2.5, 1.25, and 0.625‰) of ME against *C. violaceum* ATCC31532. A slight difference was first observed between the control group and treatment groups (sub-MIC ME) before 12 h. In the later growth stage, there was no significant distinction between ME-containing cultures and control group (Figure 1A). Measurement of CFU at 12 and 24 h showed that no differences were detected in treatment groups and the control group (Figure 1B). As expected, the growth curve and CFU results of cultures containing the ME showed no growth inhibition when compared with the control group under the conditions tested. We therefore assessed the specific effects of sub-MIC ME (5, 2.5, 1.25, and 0.625‰) on QS in *C. violaceum*.

### Violacein detection in *Chomobacterium violaceum*

**FIGURE 2:**
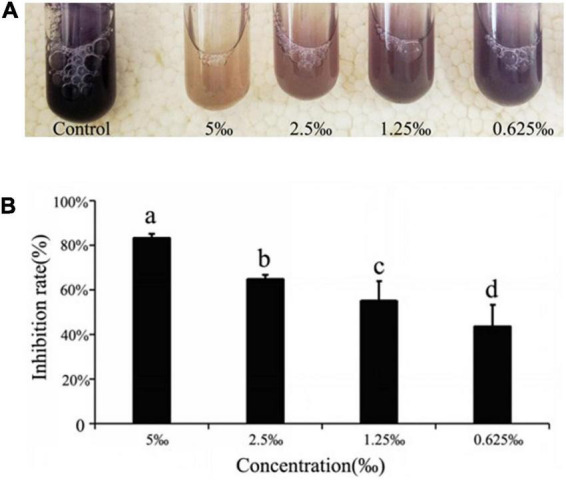
Quorum sensing (QS) inhibitory activity of ME against violacein production in *Chomobacterium violaceum* ATCC31532. **(A)** Effect of methyleugenol (ME) on violacein production in *C. violaceum*. ME-treated cultures showing progressive reduction at the concentration of sub-MIC (5, 2.5, 1.25, and 0.625‰). **(B)** Quantitative analysis of violacein inhibition rate in *C. violaceum* by ME. Mean values of triplicate independent experiments and SD were shown. Significant at *P* < 0.05.

The addition of ME was shown to affect production of the QS-regulated chromogenic toxin, violacein, the presence of which was indicated by the purple coloration of the culture medium. In assay, we observed that the addition of ME showed a visible and concentration-dependent inhibition in violacein production (Figure 2A). The purification and quantitative of the violacein in the culture supernatant again demonstrated the inhibitory effect of ME on violacein production, and the violacein inhibition showed the maximum inhibition in *C. violaceum* when treated with ME at 5‰ (the highest tested concentration) (Figure 2B).

### Effect of methyleugenol on biofilm

**FIGURE 3:**
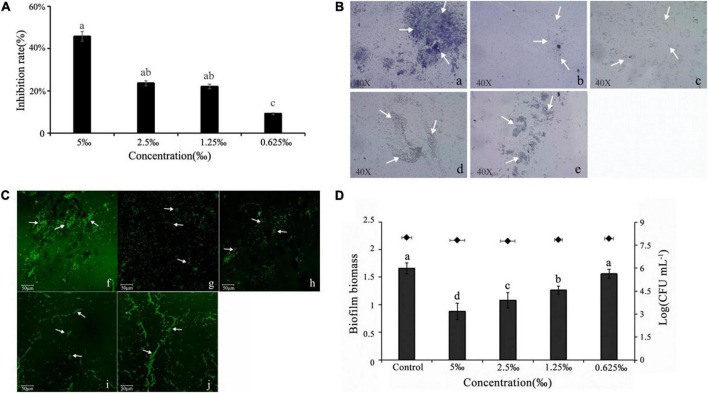
Methyleugenol (ME)-reduced biofilm formation of *Chomobacterium violaceum*. **(A)** Quantitative assessment of biofilm biomass inhibition, **(B)** light microscopic images **(a–e)**, under a light microscope at a magnification of 40×, and **(C)** confocal laser scanning microscopy (CLSM) **(f–J)**. Images of untreated and ME-treated biofilm of *C. violaceum*, **(a,f)** untreated; **(b,g)** 5‰; **(c,h)** 2.5‰; **(d,i)** 1.25‰; **(e,j)** 0.625‰. **(D)** Effect of ME on performed biofilm. The arrows are the dyed biofilm. Mean values of eight times independent experiments and SD were shown. Significant at *P* < 0.05.

Recently, a study demonstrated that the ability to form structured bacteria biofilm is linked to drug-resistance and persistence biofilm formation increases its capacity to remain in bacterial infection (Rasamiravaka et al., 2017). The anti-biofilm ability of ME against the bacterium was visible through biofilm staining with CV, and a significant reduction in biofilm biomass was observed at sub-MIC (5, 2.5, 1.25, and 0.625‰) ME by comparison with the control group (Figure 3A). In addition, ME was found to be influenced by the biofilm architecture of *C. violaceum*. The observation assay by the light microscope and the confocal laser scanning microscope indicated the well-organized disruption in biofilm architecture, treated at different concentrations of ME (Figures 3B,C).

As shown in Figure 3D, approximately 47.17% of performed biofilms were removed after treatment with 5‰ ME (the highest treated concentration). Cell survival indicated that ME at treated concentrations showed no effect on the viability of planktonic cells in the culture supernatant.

### Swarming motility

**FIGURE 4:**
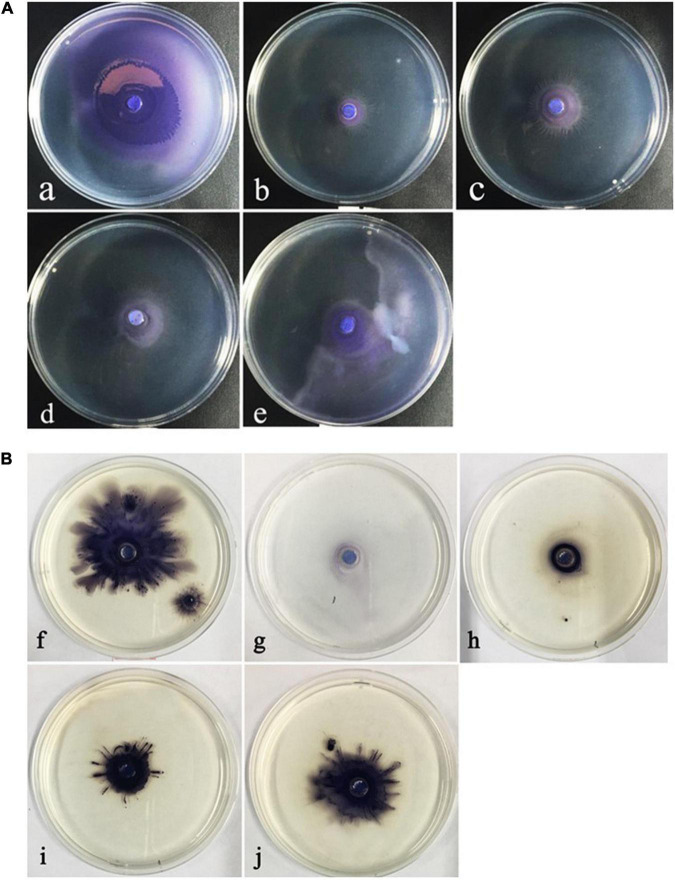
The inhibitory of the motility behavior of *Chomobacterium violaceum* by methyleugenol (ME). **(A)** Swarming **(a–e)**, **(B)** Swimming **(f–j)**, **(a,f)** control, untreated with ME. **(b–e,g–j)** treated with the concentration of sub-MIC (5, 2.5, 1.25, and 0.625‰).

The ability of motility movement plays an important role in the preliminary stage of QS-regulated bacterial biofilm formation. The obtained results showed that ME regulated the swarming and swimming motility behavior of *C. violaceum*. A visible inhibition could be observed in swarming and swimming medium, and the maximum inhibition was both found at the highest tested concentration (5‰) (Figures 4A,B).

### Detection of C6-HSL by gas chromatograohy and biosensor CV026

**FIGURE 5:**
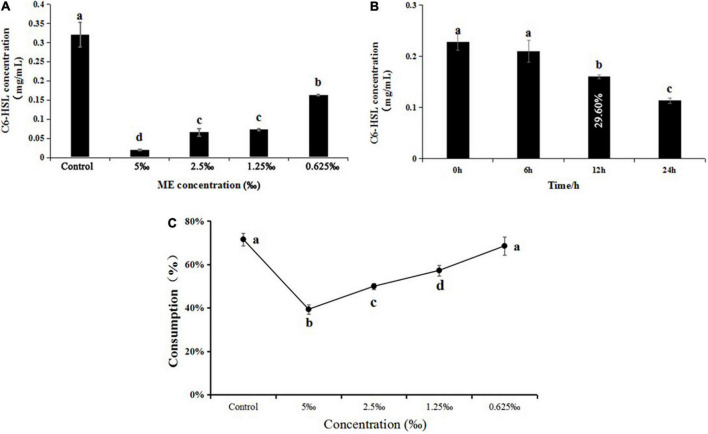
**(A)** Effect of methyleugenol (ME) at different concentration (5, 2.5, 1.25, and 0.625‰) on C6-HSL of *Chomobacterium violaceum*. **(B)** Effect of ME (5‰) on C6-HSL treated with 0, 6, 12, and 24 h. **(C)** Effect of ME at different concentration (5, 2.5, 1.25, and 0.625‰) on C6-HSL of *C. violaceum* CV026. The results were the mean (*n* = 3) ± standard deviation. Values followed by different letters indicate significant difference at *P* < 0.05.

The C6-HSL extracts of *C. violaceum* exposed to ME (5, 2.5, 1.25, and 0.625‰) induced a lower level of purple pigment in biosensor CV026 as compared to control in a semi-quantitative assay (Supplementary Figure 1). In the quantitative assay, the retention time and standard curve of C6-HSL can be found in the previous study (Wang et al., 2019a). In line with CV026 results, the concentration of C6-HSL in *C. violaceum* exposed to ME obviously decreased by comparison with the control, and the concentrations were 0.32, 0.02, 0.06, 0.07, and 0.16 mg/ml, respectively (Figure 5A and Supplementary Figure 2). The capacity of ME targeted directly at C6-HSL was detected by adding exogenous C6-HSL to LB broth containing ME (the highest tested concentration of 5‰). Compared to control, the C6-HSL extract had a slight change at 6 h and subsequently showed a significant drop in C6-HSL content with the extension of incubation time (at 12 and 24 h) (Figure 5B and Supplementary Figure 3), suggesting that ME could degrade the C6-HSL directly.

Furthermore, the C6-HSL extracts from *C. violaceum* CV026 treated with ME (5, 2.5, 1.25, and 0.625‰) showed that the consumption of exogenous C6-HSL was lowest in the presence of the highest tested concentration (5‰) (Figure 5C and Supplementary Figure 4), indicating that ME might be able to interact with the CviR protein.

### Expression of the Quorum sensing-related gene to response methyleugenol

**FIGURE 6:**
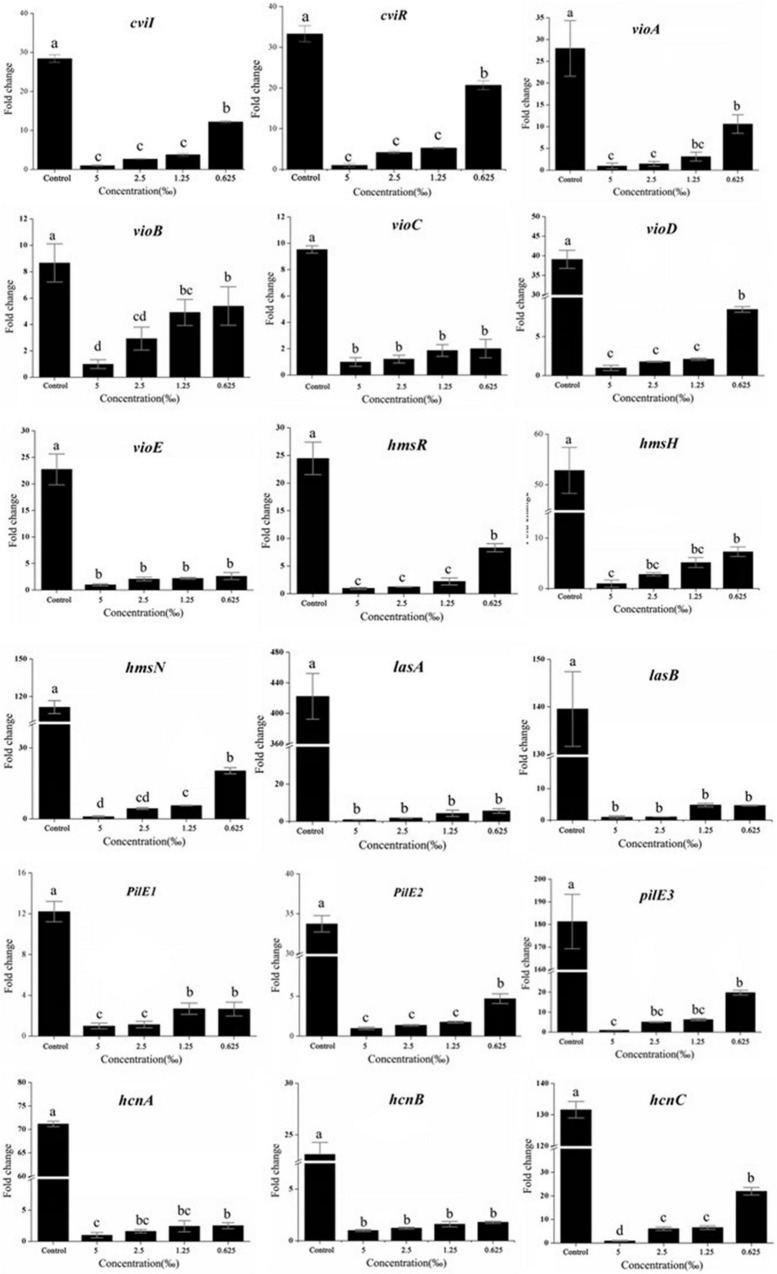
Effect of methyleugenol (ME) on the expression of genes QS-regulated. The *cviI*, *cviR*, *vi-oABCDE*, *hmsHRN*, *lasAB*, *pilE1-3*, and *hcnABC* were detected in response to ME treatment. Expression of the house-keeping gene, *rpoD*, was used as the internal control for each sample. The ME concentration of treatment was as follows: 5, 2.5, 1.25, and 0.625‰. Control was untreated.

To investigate the effect of ME on the QS system of LuxI/LuxR, RT-qPCR was performed to detect the expression of QS-system genes. As expected, the expression of *cviI* and *cviR* genes, the LuxI/LuxR homologous, was downregulated, and both had a dose-dependent reduction in response to the different concentrations of ME (Figure 6).

A number of *C. violaceum* phenotypic characteristics, regulated by and associated with QS, have been revealed in the complete genome of *C. violaceum*, reported in 2003 (de Almeida et al., 2003). Thus, we detected the effect of ME on the expression of important virulence factors, including violacein production (*via vioA-E*) (Duran et al., 2016), the biofilm formation (*via hmsHNFR*), elastase production (*via lasA* and *lasB*) (Zins et al., 2001), pilus (*pilE1-3*) (de Oca-Mejía et al., 2015), and cyanide production (*hcnA-C*) (Rodgers and Knowles, 1978). Excepting *hmsF* (Supplementary Figure 5), other genes (*vioA-E*, *hmsHNR*, *lasA and lasB*, *pilE1-3*, and *hcnA-C*) were obviously repressed in the presence of ME (sub-MIC, 5, 2.5, 1.25, and 0.625‰) (Figure 6). Our findings suggest that ME can be used to significantly inhibit the key virulence factors of *C. violaceum* without having any direct effect on growth rate.

### Molecule docking

**FIGURE 7:**
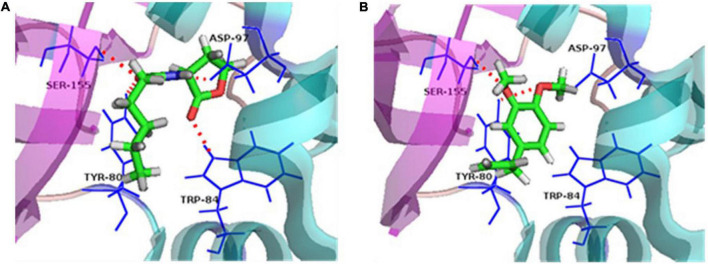
Ligand-protein interaction. **(A)** Docking of C6-HSL and CviR protein. **(B)** Docking of methyleugenol (ME) and CviR protein. Target structures were shown as ribbon diagram. The ligand structures are colored in green, red, and gray. Red dashed lines represent the hydrogen bonds.

Molecule docking methodology, which explores the behavior of small molecules in the binding site of a target protein, is intensively applied in the anti-QS research (Wu et al., 2014; Pagadala et al., 2017). The piroxicam and meloxicam (Soheili et al., 2015), sodium houttuyfonate (Wu et al., 2014), eugenol and carvacrol (Joshi et al., 2016b), and trans-cinnamaldehyde (Chang et al., 2014) were found to possess anti-QS activities by directly binding to the homolog of LuxR and/or LuxI by molecules docking. In our research, docking results showed that the binding energies between the receptor proteins, CviR and C6-HSL/ME, were –6.7 and –6.2 kcal/mol, exhibiting similar affinity. The natural ligand of CviR, C6-HSL, was predicted to make hydrogen bonds with Tyr80, Trp84, Asp97, and Ser155 in the CviR activity site (Figure 7A). The methyleugenol formed two hydrogen bonds with binding site residues Try80 and Ser155 in the CviR activity site (Figure 7B), suggesting a probable interaction with the CviR protein. Except for the H-bonds interaction, the residues including Leu85, Tyr88, Ile99, Leu100, Trp111, Phe115, and Ile153 in the CviR structure can form hydrophobic interactions with the C6-HSL, while the test molecule, ME, can interact with the residues including Leu72, Val75, Leu85, and Tyr88 through hydrophobic interaction. Combined with the H-bonds interaction, these results showed that CviR had a stronger interaction with the control C6-HSL when compared with the ME molecule.

## Discussion

Bacterial infections are a global public health concern and one leading cause of this problem is the pathogens’ drug-resistance, which involves the administration of multiple drugs and antibiotics to improve the therapeutic effects (Pimenta et al., 2014). From an application perspective, the compounds isolated from natural products will be excellent alternatives to chemical agents and antibiotics in reducing the risk of drug-resistance and bacterial infections. In this study, anti-QS activity of ME was evaluated against the *C. violaceum* ATCC31532.

Initially, the inhibitory activities of inhibitors were quantified by measurement of the production of related virulence factors. Unlike antibiotics that are used to inhibit bacterial cell growth, it is important to identify QS-inhibitors that can inhibit pathogenicity without allowing bacteria to develop drug-resistance. Present studies revealed that ME, isolated from *M. bracteata* EO, had a dramatic reduction in the violacein production and biofilm biomass at sub-inhibitory concentrations (sub-MICs) (Figures 2, 3). Essential oil from *Ferula* (Wang et al., 2016) and tannin-rich fraction from *Terminalia catappa* (Taganna et al., 2011) revealed the similar function. Biofilm formation is known to be tightly associated with QS system in several pathogens including *C. violaceum* (Vasavi et al., 2014). Biofilm formation is closely related to bacterial infection that attached to the surfaces of microbial communities by polysaccharides, protein, and nucleic acids. Once biofilms are formed, the bacteria in the biofilm structure are from 10 to 1,000 times more resistant to antimicrobial and are difficult to remove, being less susceptible to host defenses and antibacterial agents resulting in the persistent infections (Lee et al., 2014; Mon et al., 2015), thus interfering with the biofilm have enormous impact to attenuate the virulence of diseased pathogens and drug-resistance. Here, *C. violaceum* formed biofilms were observed using the light microscope and the confocal laser scanning microscope, indicating the obvious disruption in biofilm architecture in the presence of ME. It was seen that microscopic observation matched the quantitative biofilm biomass results (Figures 3B,C). Generally, flagella-based motility is considered important factors for the maturation of biofilm structure and the enhancement of QS (Bak et al., 2015; de Oca-Mejía et al., 2015). In line with the previous studies, swarming and swimming movement in *C. violaceum* had an apparent inhibition when treated with ME (Figures 4A,B), partially resulting in the reduction of the biofilm biomass and disruption of biofilm architecture in *C. violaceum*. To further investigate the molecular mechanism responsible for QS inhibition, RT-qPCR was used to determine the expression level of QS system (*cviI*, *cviR*) and virulence genes (*hmsF*HNR, vioABCDE, *lasA*B, *hcnABC*, and *pilE1-3*) in *C. violaceum*. Except *hmsF*, the RT-qPCR results were well-matched the biofilm quantification result, thus we presumed that *hmsF* were not regulated by QS system solely or directly, thus not downregulated by ME treatment.

The QS system was first described in the marine luminescent bacterium *Vibro ficheri*, which controls transcription of the luminescence (lux) operon, and LuxI synthase and LuxR transcriptional activator constitute this QS system (Antunes et al., 2007). LuxR as a dimer with the N-terminal binding to its pheromone and the C-terminal binding to the targeted DNA domain is responsible for lux gene activation. In the absence of AIs, the N-terminal domain blocks the function of C-terminal domain (Chong et al., 2011). At a sufficiently high AIs concentration, AIs is bound to the N-terminal domain and LuxR binds to the lux box and subsequently activates transcription of the lux operon (*luxICDABEG*) (Egland and Greenberg, 2001; Castang et al., 2006). Thus, AI production is a vital indicator in the process of interfering with the QS system and eliminating the virulence factor. In our study, we first detected the effect of ME on C6-HSL production in *C. violaceum*, GC quantitative assay revealed that the C6-HSL production was obviously reduced when treated with ME (sub-MIC) for 12 h (Figure 5A), as revealed by the biosensor CV026 (Supplementary Figure 1). In addition, we found that the exogenous C6-HSL in LB broth degraded approximately 29.60% in the presence of 5‰ ME for 12 h (Figure 5B). These data suggested that the inhibitory effect of ME on the C6-HSL production was mainly responsible for the reduction of C6-HSL in *C. violaceum*. Collectively, the findings presented in this study indicated that ME interfered directly with the QS machinery in *C. violaceum*.

**FIGURE 8:**
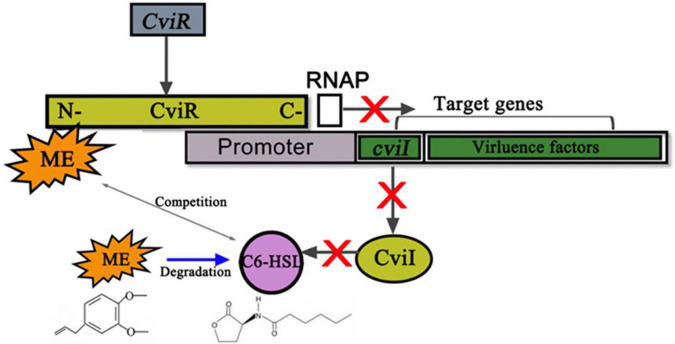
Putative mechanism for the inhibition of *Chomobacterium violaceum* Quorum sensing (QS) by methyleugenol (ME). ME may complete with C6-HSL for binding CviR to reduce the enzyme activity of cviR (gray arrow between ME and C6-HSL). Subsequent *cviI* and virulence factors, which positively regulated by *cviR*, were repressed by ME. The RNAP represents the RNA polymerase. Meanwhile, the gray arrows stand for the positive regulatory. Blue arrow between ME and C6-HSL meant that ME was able to degrade the C6-HSL. Red X marks represent the block function.

Besides, the change of exogenous C6-HSL in CV026 proved that ME may interact with the CviR protein, while ME was unlikely to interfere with the synthesis of autoinducers in CV026 because CV026 had a defective luxI synthase gene. At the same time, ME was docked into the potential binding site of the CviR model (Figure 7B), supporting a direct competition between ME and endogenous ligands (C6-HSL). Recently, the potential interaction of compounds with LuxR/LuxI homologous protein has been studied at the atomic level and experimental level. Swem et al. (2009) revealed that CTL (Chloro thiolactine) and CL (Chloro lactone) bound to the CviR AHL receptor and acted as potent antagonists. Packiavathy et al. (2012) found that four compounds, including ME, lauric acid, linalool, and capric acid, displayed significant antagonistic activity against LasR receptor protein through molecular docking analysis. Joshi et al. (2016b) discovered that eugenol and carvacrol can dock into the potential binding sites of ExpI and ExpR models and scored better than the known inhibitors. Understanding the molecular mechanism at the atomic level will undoubtedly facilitate the excavation of new QS-inhibitors. Nevertheless, there was no direct research to clarify the molecular mechanism of the inhibitory effects of QSI on the QS system. Here, the results of this study strengthened the hypothesis by which ME indeed exerted its effect by directly binding to cviR protein, and the putative mechanism for the inhibition of *C. violaceum* QS by methyleugenol was also drawn (Figure 8).

On the background of unexpected bacterial infection outbreaks, novel approaches and natural products that are thought safe to hold great potential for preventing and controlling bacterial contamination. Our results strongly suggested that the methyleugenol (ME), the principal active components of *M. bracteata* EO against bacterial QS activity, could be a candidate source of QS-inhibitors. Meanwhile, the anti-QS mechanism of ME consisted of inhibition of C6-HSL production, potentially *via* interaction with CviR and/or CviI protein(s). More studies are still clearly required to ascertain the exact binding sites and mechanism actions.

## Conclusion

Current studies have clearly demonstrated that the ME isolated from *M. bracteaca* EO can inhibit virulence (violacein production, biofilm formation, and motility movement), C6-HSL production, and QS-related genes expression of *C. violaceum*; its anti-virulence activity may prove to be a new approach to prevent pathogens contamination. In addition, a preliminary study on the mechanism of inhibition of *C. violaceum* by ME laid the foundation for further studies to confirm this effect at the molecular levels.

## Data availability statement

The original contributions presented in this study are included in the article/Supplementary material, further inquiries can be directed to the corresponding authors.

## Author contributions

WW: conceptualization, methodology, software, validation, formal analysis, writing—original draft preparation, and writing—review and editing. XL: validation and investigation. HY, XH, LP, and CY: validation. LZ: methodology. SW: conceptualization. YL: writing—review and editing and funding. All authors agreed to be accountable for the content of the study.
