# Supplementary material for: Anti-quorum sensing evaluation of methyleugenol, the principal bioactive component, from the Melaleuca bracteata leaf oil
Source: Front Microbiol. 2022 Aug 22;13:970520. doi: 10.3389/fmicb.2022.970520 (PMC9477228; doi:10.3389/fmicb.2022.970520)
Supplement: Supplementary file 1 [file Data_Sheet_1.docx]

Supplementary Material

# Supplementary Figures


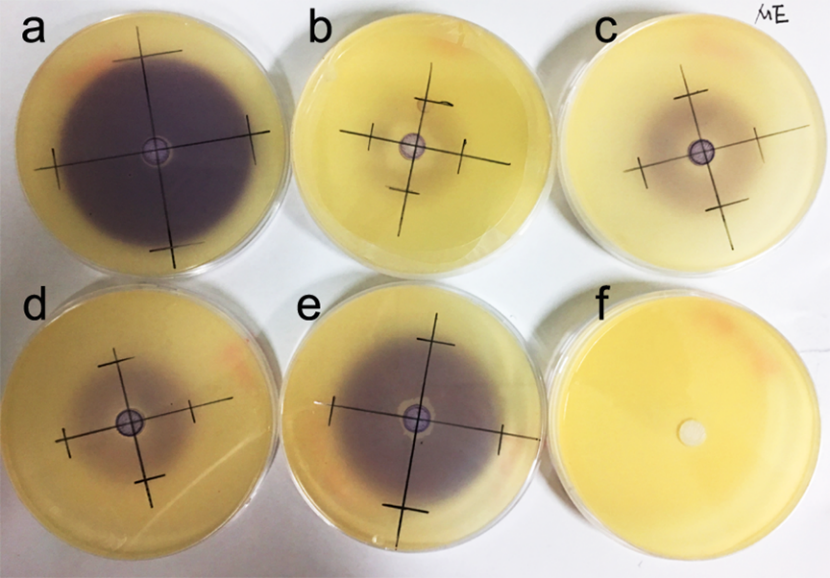


**Supplementary Figure 1.** Induction of purple color production in CV026 by supernatants of *C.violaceum* culture grown in LB untreated and treated with different concentration of methyleugenol. a: control, untreated with ME. b-e: treated with the concentration of sub-MIC (5‰, 2.5‰, 1.25‰ and 0.625‰). f: ethyl acetate.


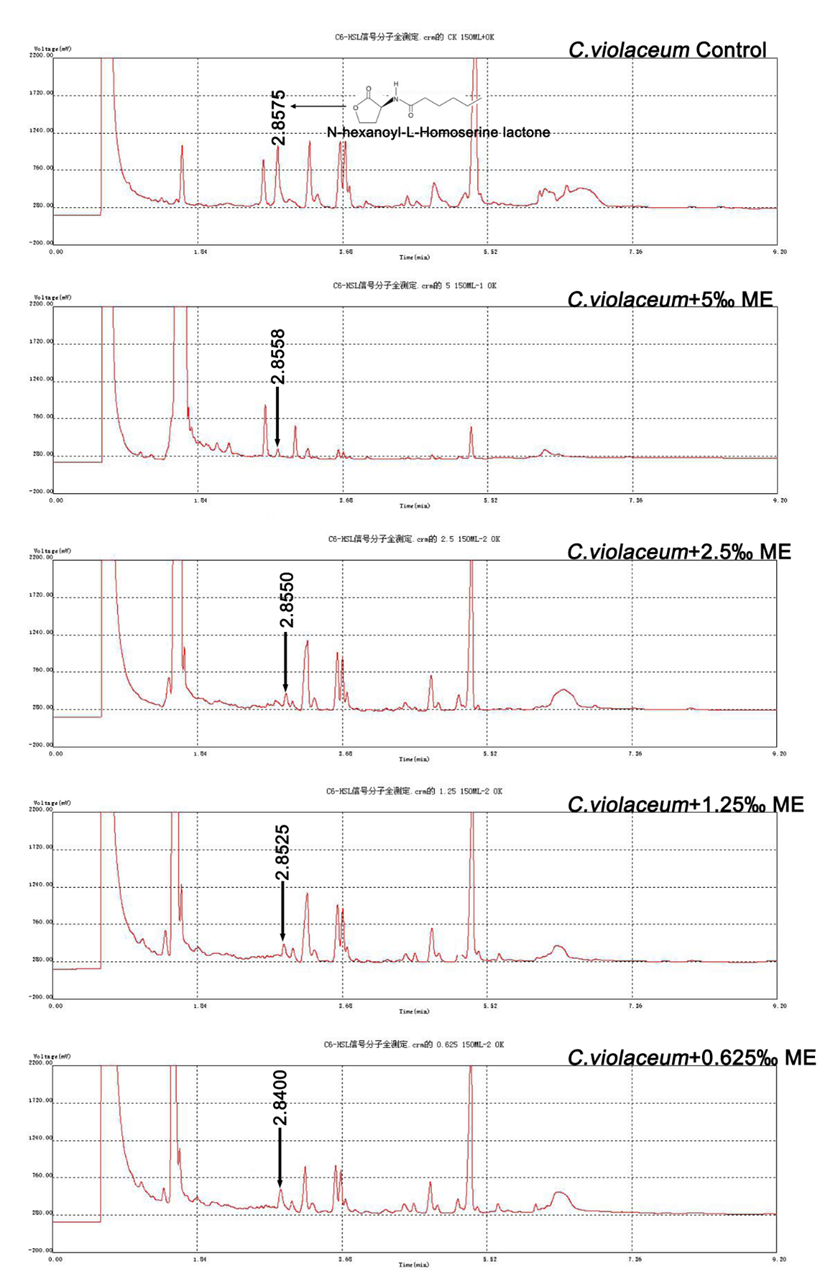


**Supplementary Figure 2.** GC analysis the effect of methyleugenol at different concentration (5‰, 2.5‰, 1.25‰ and 0.625‰) on C6-HSL production of *C.violaceum*.


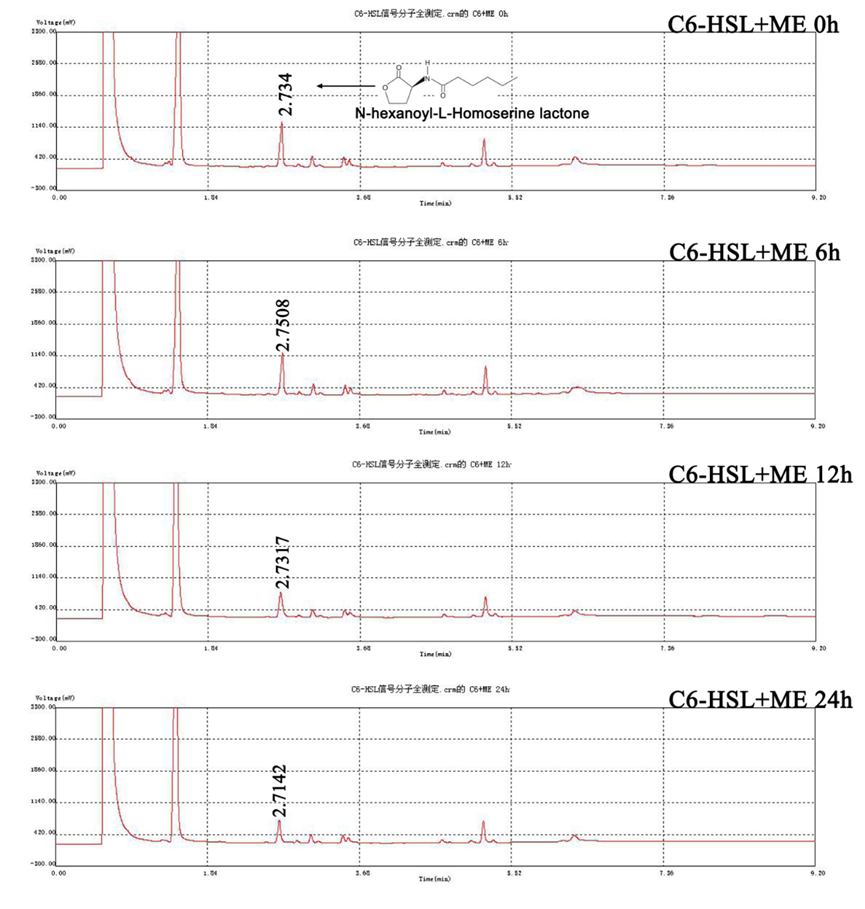


**Supplementary Figure 3.** GC analysis effect of methyleugenol (5‰) on C6-HSL treated with 0h, 6h,12h and 24h*.*


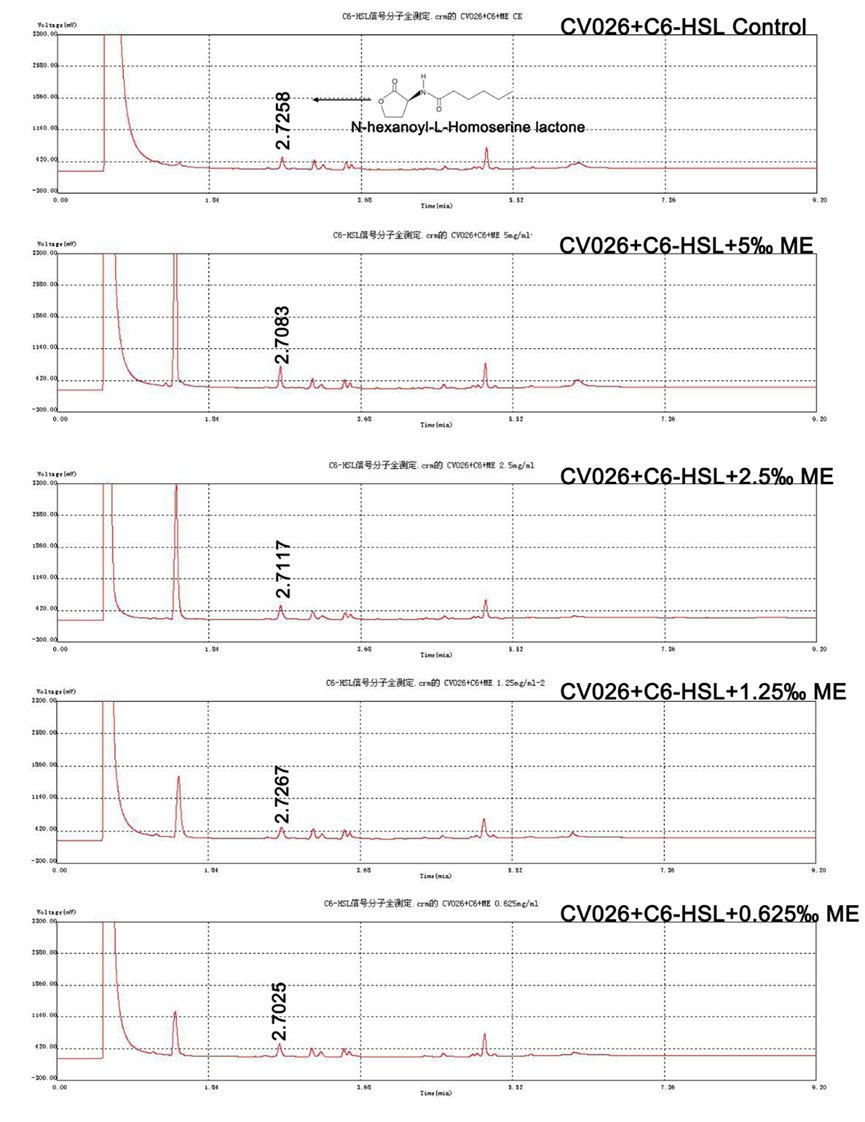


**Supplementary Figure 4.** GC analysis the effect of methyleugenol at different concentration(5‰, 2.5‰, 1.25‰ and 0.625‰) on C6-HSL of *C.violaceum* CV026.


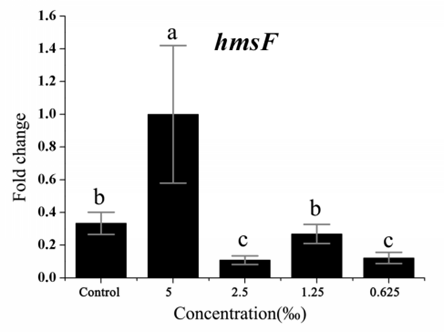


**Supplementary Figure 5.** Effect of methyleugenol on the expression of *hmsF*.
